# Probing the protective mechanism of poly-ß-hydroxybutyrate against vibriosis by using gnotobiotic *Artemia franciscana* and *Vibrio campbellii* as host-pathogen model

**DOI:** 10.1038/srep09427

**Published:** 2015-03-30

**Authors:** Kartik Baruah, Tran T. Huy, Parisa Norouzitallab, Yufeng Niu, Sanjay K. Gupta, Peter De Schryver, Peter Bossier

**Affiliations:** 1 Lab of Aquaculture & *Artemia* Reference Center, Department of Animal Production, Faculty of Bioscience Engineering, Ghent University, Rozier 44, Gent 9000, Belgium; 2 Directorate of Cold Water Fisheries Research, Chhirapani Field Centre, Champawat, Uttarakhand 262523, India

## Abstract

The compound poly-ß-hydroxybutyrate (PHB), a polymer of the short chain fatty acid ß-hydroxybutyrate, was shown to protect experimental animals against a variety of bacterial diseases, (including vibriosis in farmed aquatic animals), albeit through undefined mechanisms. Here we aimed at unraveling the underlying mechanism behind the protective effect of PHB against bacterial disease using gnotobiotically-cultured brine shrimp *Artemia franciscana* and pathogenic *Vibrio campbellii* as host-pathogen model. The gnotobiotic model system is crucial for such studies because it eliminates any possible microbial interference (naturally present in any type of aquatic environment) in these mechanistic studies and furthermore facilitates the interpretation of the results in terms of a cause effect relationship. We showed clear evidences indicating that PHB conferred protection to *Artemia* host against *V. campbellii* by a mechanism of inducing heat shock protein (Hsp) 70. Additionally, our results also showed that this salutary effect of PHB was associated with the generation of protective innate immune responses, especially the prophenoloxidase and transglutaminase immune systems – phenomena possibly mediated by PHB-induced Hsp70. From overall results, we conclude that PHB induces Hsp70 and this induced Hsp70 might contribute in part to the protection of *Artemia* against pathogenic *V. campbellii.*

According to reports by the United Nations' Food and Agriculture Organization (FAO), bacterial disease outbreaks are considered a significant constraint to the development of the aquaculture sector and the terrestrial animal production sector. The ban on the use of antibiotics to control diseases in these production sectors has challenged researchers throughout the world to look for alternative biocontrol strategies^1^,^2^. Recently, it has been suggested that short-chain fatty acids (SCFAs) could be useful as biocontrol agents to control bacterial diseases in animal production and more specifically aquaculture^3^. SCFAs, such as acetate, propionate and butyrate, are the major products of anaerobic bacterial fermentation of non-absorbable carbohydrates in the small intestine^4^. Several studies have demonstrated that SCFAs inhibit the growth of enterobacteria like *Salmonella typhimurium, Escherichia coli* and *Shigella flexneri*^5^. Recently, luminescent vibrios like *Vibrio campbellii* were also reported to be as susceptible to SCFAs^6^. Furthermore, *in vivo* challenge tests exhibited that SCFAs significantly increased the survival of the aquaculture model organism *Artemia franciscana* larvae challenged with pathogenic *Vibrio campbellii*, indicating that these compounds are not only useful in terrestrial animal production, but also in aquaculture^6^. In another report, the well-known bacterial storage compound poly-ß-hydroxybutyrate (PHB), a polymer of the SCFA ß-hydroxybutyrate, was also shown to protect *Artemia* larvae from the virulent *V. campbellii* strain^7^. Although this compound is insoluble in water, it is likely to be biologically degraded into antibacterial β-hydroxybutyric acid or PHB oligomers in the gastrointestinal tract of organisms^7^. The degraded product can exert its beneficial effects like other SCFA do^7^. In several experiments with *A. fransiscana*, this approach increased the survival of the animals with up to 73% upon challenge with the pathogen *V. campbellii*^7^,^8^.

It has also previously been shown that the SCFA butyrate induces heat shock protein (Hsp) 25 in rat intestinal epithelial cells and protects the latter against oxidant injury^9^. Hsps are a group of highly conserved proteins of which expression is constitutive or inducible under different conditions. Hsps, particularly Hsp70, have strong cytoprotective effects and behave as molecular chaperones for maintaining proper protein folding, disaggregating, and refolding misfolded protein as well as targeting damage proteins for degradation^10^,^11^. Besides these, Hsp70 also generate protective immunity against many diseases as demonstrated in a wide variety of animal models^12^,^13^,^14^. For instance, in an axenically hatched *A. franciscana*, it was well demonstrated that upregulation of endogenous Hsp70 by a Hsp-inducing compound^15^ or exogenous administration of *Artemia* Hsp70 or the *Escherichia coli* Hsp70 equivalent DnaK, protected *Artemia* against *V. campbellii* infection by priming the prophenoloxidase system^16^,^17^. Similar results were also obtained by Ryckaert et al. (2009) for platyfish *Xiphophorus maculates* injected with Hsp70 protein and challenged with pathogenic bacteria *Yersinia ruckeri*.

This study addresses the possibility that the protective effects of PHB could be related to induction of Hsp70. Here, using the *Artemia*-*V. campbellii* host-pathogen model, we present novel findings which demonstrate that PHB is indeed a potent *in vivo* enhancer of Hsp70 and this effect mediates, at least in part, the PHB-induced protection to *V. campbellii*-challenged *Artemia*.

## Results

### PHB confers protection to *Artemia* against pathogenic *V. campbellii*

**Figure 1:**
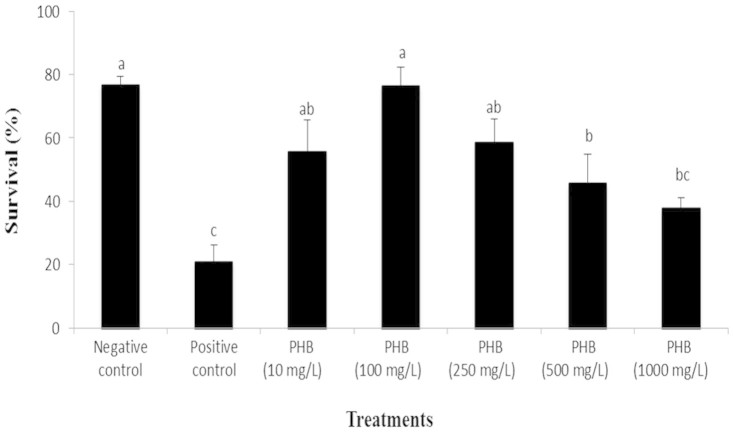
PHB at an optimum concentration confers protection to *Artemia* larvae against *V. campbellii*. PHB particles were added to the *Artemia* culture water at indicated concentrations. Simultaneously, the larvae were fed with autoclaved LVS3 and challenged with *V. campbellii*, each at 10^7^ cells/mL, for 48 h. PHB-untreated *Artemia* challenged with *V. campbellii* (positive control) and those not challenged with *V. campbellii* (negative control) served as controls. Values are presented as mean ± standard error and representative of 3 independent experiments. Different letters indicate significant differences (P < 0.05).

In a previous study, PHB particles (1000 mg/L) with an average diameter of 30 µm were shown to confer protection to *Artemia* against *V. campbellii*^7^. To ascertain the protective effect of PHB and also to re-optimize the effective dose of PHB particles of size 25–30 µm, we carried out an *in vivo* survival assay using a range of PHB doses from 0 to 1000 mg/L. As shown in Figure 1, PHB significantly enhanced the survival of the challenged *Artemia* when added at a concentration of 10 (2.6-fold increase), 100 (3.6-fold increase), 250 (2.8-fold increase) and 500 mg/L (2.2-fold increase). A complete protection (no significant differences in survival with negative control) was observed at 100 mg/L concentration. Increasing the PHB concentration to 1000 mg/L did not further increase the survival of the larvae. In contrast, the survival decreased markedly, reaching the level of the negative control.

### PHB induces Hsp70 production in *Artemia*

**Figure 2:**
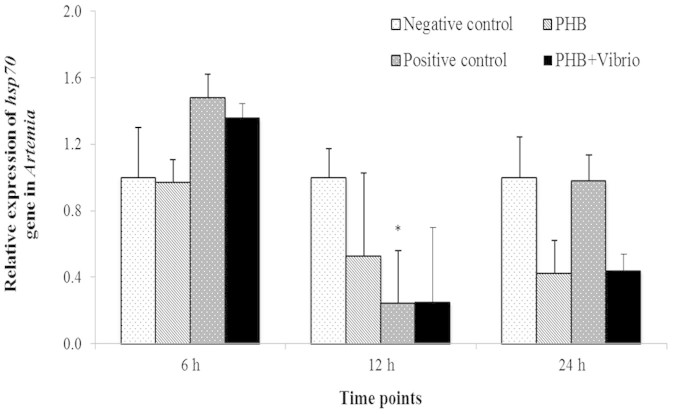
PHB at an optimum concentration regulates the expression of *hsp70* gene in *Artemia* larvae challenged with *V. campbellii*. PHB particles (100 mg/L) were added to the *Artemia* culture water at the start of the experiment. Simultaneously, the larvae were fed with autoclaved LVS3 and either challenged with *V. campbellii* (PHB + *Vibrio*) at indicated concentrations or not (PHB). Controls were maintained as described in Figure 1. Samples were collected for *hsp70* gene expression assay at 6, 12 and 24 h of *Vibrio* challenge. The expression of *hsp70* gene in the negative control was regarded as 1.0. Results, which are the mean of 3 replicates, are presented relative to *Artemia* actin gene expression, according to the equation of Pfaffl et al. (2002). Bars indicate standard error from the mean. Significant differences between the treatment and (negative) control at corresponding time points are indicated by *(P < 0.05).

**Figure 3:**
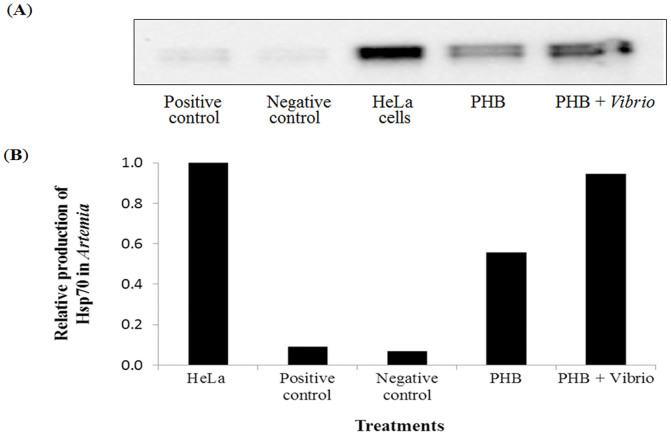
PHB at an optimum concentration induces Hsp70 production in *Artemia* larvae challenged with *V. campbellii*. For the treatment groups, refer to Fig. 2. for explanation. *Artemia* samples were collected for analysis of Hsp70 induction at 6 h of *Vibrio* challenge. (A) Protein extracted from different groups was resolved in SDS-PAGE gel and then transferred to polyvinylidene fluoride membranes and probed with antibody to *Artemia* Hsp70. Seven microgram of *Artemia* protein was loaded in each lane. HeLa (heat shocked) cells (6 µg) were loaded on to one well to serve as a positive technical control and for calculating the relative amount of Hsp70 in the sample. Cropped blot image was shown and the full-length blot was presented in Supplementary Figure S1. (B) Quantitative analysis of Hsp70 in the *Artemia* larvae (expressed relative to the amount in HeLa cells).

To determine whether induction of Hsp70 may be a mechanism for increased resistance of PHB-treated *Artemia* against *V. campbellii* challenge, we analyzed the temporal induction profile of Hsp70 by two approaches: qPCR analysis to determine the expression of mRNA for inducible *hsp70*, and immunoblotting to analyze Hsc70 and Hsp70 proteins. The results of qPCR revealed that at 6 h post treatment, the expression of the *hsp70* gene in the PHB and PHB + *Vibrio* groups did not increase significantly as compared to the negative control (P > 0.05, Figure 2). At 12 and 24 h post treatment, the *hsp70* mRNA expression levels in the PHB and PHB + *Vibrio* groups appeared to decrease compared to the negative control, however, no significant difference was observed among the groups. The expression pattern of *hsp70* gene in *Artemia* challenged with *V. campbellii* (positive control), relative to the unchallenged (negative) control, tend to increase at 6 h post challenge (P > 0.05), then significantly decreased by 4-fold at 12 h post challenge (P < 0.05). However, at 24 h post challenge, the *hsp70* transcripts increased to the same level as that of the control.The Western blot analysis showed that PHB markedly increased Hsp70 production in the *Vibrio*-challenged and unchallenged *Artemia* (by 10.5 and 6.2-fold, respectively) when compared with the (negative) control at 6 h post treatment (Figure 3). At 12 and 24 h post treatment, Hsp70 was not detected in the PHB-treated groups (data not shown).

### PHB regulates the expression of innate immune-related genes in *Artemia*

**Figure 4:**
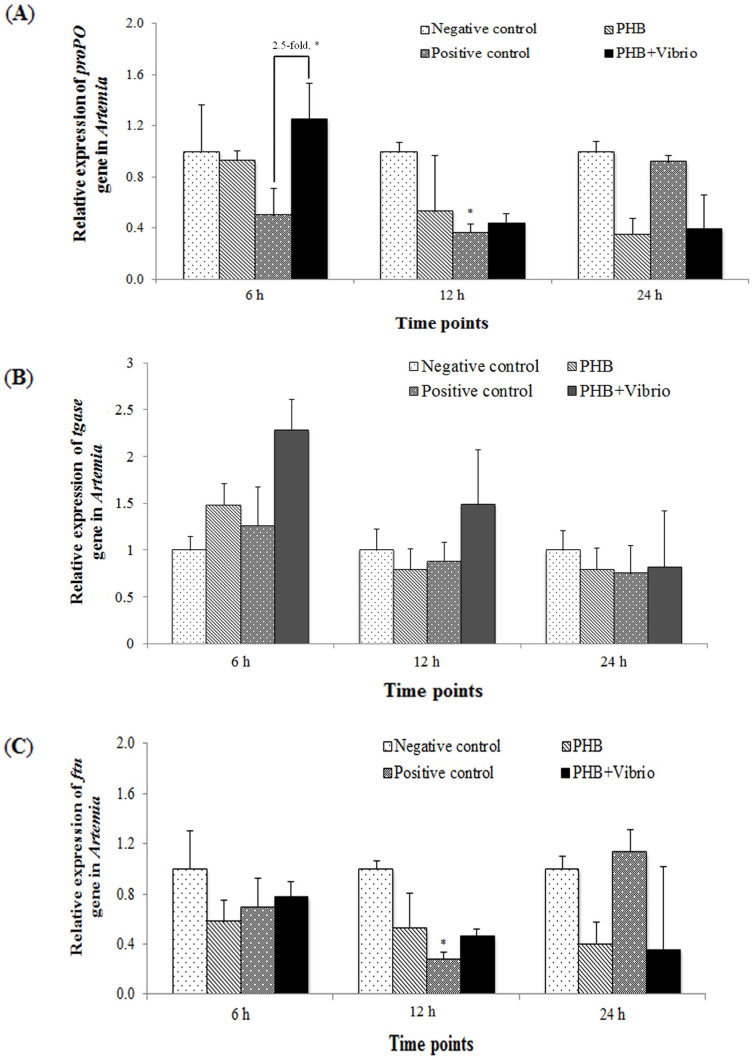
PHB regulates the expression of (A) *proPO* (B) *tgase* and (C) *ftn* genes in *Artemia* larvae challenged with *V. campbellii*. For the treatment groups, refer to Fig. 2. for explanation The expression of the *proPO*, *tgase* and *tgase* gene in the negative control was regarded as 1.0. Results, which are the mean of 3 replicates, are presented relative to *Artemia* actin gene expression, according to the equation of Pfaffl et al. (2002). Bars indicate standard error from the mean. Significant differences between the treatment and untreated control at corresponding time points are indicated by * (P < 0.05).

Since there exists a correlation between the amount of induced Hsp70 and the degree of improved protective immune responses against diseases in animals^19^,^20^, we next determined the temporal expression of the innate immunity-related genes i.e. prophenoloxidase (*proPO*), transglutaminase (*tgase*) and ferritin (*ftn*) in *Artemia* treated with PHB and simultaneously challenged with *V. campbellii*. As shown in Figure 4A, there was no significant upregulation of the *proPO* gene in the PHB-treated groups (PHB and PHB + *Vibrio*) as compared to the unchallenged (negative) control at any of the time points tested. The expression pattern of the *proPO* gene in *Artemia* challenged with *V. campbellii* (positive control) showed a different trend. The expression levels, relative to that in the negative control, tend to decrease at 6 h post challenge (P > 0.05), significantly down regulated at 12 h post challenge (P < 0.05), and then returned to control level at 24 h post challenge (P > 0.05). We also compared the expression level of the *proPO* gene between challenged *Artemia* (positive control) and PHB-treated challenged *Artemia* (PHB + *Vibrio*) at all the time points tested. The *proPO* expression level in the PHB + *Vibrio* group was significantly higher (2.7-fold, P < 0.05) than the positive control at 6 h post challenge and then remained unaltered at 12 h and 24 h post challenge.

The mRNA transcript levels of *tgase* in the PHB + *Vibrio* group markedly increased by 2.3-fold (P > 0.05) relative to the negative control at 6 h post treatment (Figure 4B). At 12 h post challenge, the transcript levels in this group tend to remain relatively higher (by 1.5 fold, P > 0.05) than that in the negative control. At 24 h post challenge, the transcript level was comparable with the negative control. Relative to the positive control, the *tgase* expression level in the PHB + *Vibrio* group remained considerably higher at 6 h (by 1.7-fold) and 12 h (1.4-fold) post challenge, but was not significantly different. At 24 h post challenge, no significant difference was noted between the two groups.

The *ftn* mRNA transcript levels in the PHB and PHB + *Vibrio* groups did not increase significantly as compared to the negative control, at any of the time points tested, however the expression level appeared to decrease over time (P > 0.05; Figure 4C). The expression pattern of *ftn* gene in the *Artemia* challenged with *V. campbellii* (positive control), relative to the unchallenged (negative) control, remained at the same level at 6 h post challenge and then significantly decreased by 3.6-fold at 12 h post challenge (P < 0.05). However, the *ftn* transcripts increased to the same level as that of the negative control at 24 h post challenge. In relative to positive control, the level of *ftn* transcripts in the PHB + *Vibrio* group remained unaltered at 6 and 12 h of *Vibrio* challenge. However, at 24 h, the level appeared to decrease as compared to the positive control (P > 0.05).

### PHB increases the activity of phenoloxidase in *Artemia* challenged with *V. campbellii*

**Figure 5:**
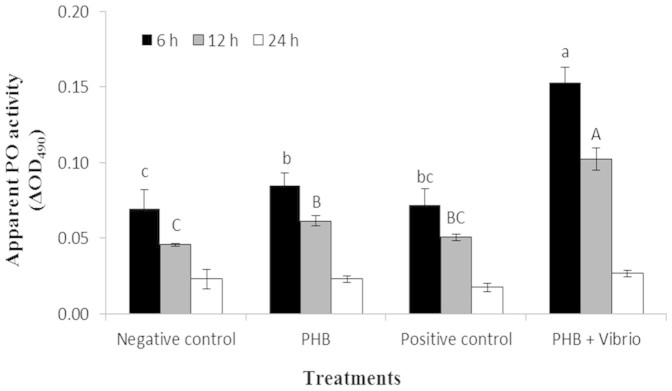
PHB increases the activity of phenoloxidase in *Artemia* challenged with *V. campbellii*. For the treatment groups, refer to Fig. 2 for explanation. *Artemia* samples were collected for the PO assay after 6, 12 and 24 h of *Vibrio* challenge. All treatments were carried out in three replicates. Values are presented as mean ± standard error (n = 3). Different letters (small and capital letters for 6 and 12 h, respectively) indicate significant differences among the groups (P < 0.05). Bars (at 12 h) with no letters indicate no significant differences (P > 0.05).

Besides measuring *proPO* at the transcriptional level, the assay of proPO at the protein level was also carried out in *Artemia* that were treated and challenged with PHB and/or *V. campbellii* for 24 h (Figure 5). The activity of phenoloxidase (PO) in challenged *Artemia* was significantly enhanced by PHB at both 6 and 12 h post challenge, where an increase of about 2-fold was seen over challenged-*Artemia* (positive control). The PO activity in PHB-treated *Artemia* was also significantly higher than that of the untreated *Artemia* (negative control). However, when compared with *Vibrio*-challenged *Artemia*, the PO activity in the PHB-treated *Artemia* was not significantly different. At 24 h post challenge, no significant differences in the PO activity level were observed among the different groups (P > 0.05).

## Discussion

The SCFA ß-hydroxybutyrate and its polymer PHB have in a number of model organisms been shown to induce protective effects against pathogenic stressors resulting in increased survival^3^,^21^. Consistent with the previous report^7^, our results provided clear evidence that addition of PHB particles to the *Artemia* culture water significantly protected the shrimp against *V. campbellii*, but significant differences between that study and the current one existed in relation to the effective PHB dose. Maximum and complete protection against *V. campbellii* was obtained at a PHB concentration of 1000 mg/L in the previous study, whereas in this study, only 100 mg/L was needed to obtain a similar effect. This disparity in the effective doses between the two studies could be attributed to the particle size of the PHB. The particles used in this study are in the range of 25–30 µm (compared to an average diameter size of 30 µM in the earlier study). The brine shrimp *Artemia* is a continuous, non-selective particle-filtration feeder but particle size has been shown to affect uptake efficiency^22^. The uptake of the smaller sized particles in this study might have been more efficient resulting in a complete protective effect being obtained at 10 times lesser dose.

The protective mechanisms of PHB and/or its degradation product ß-hydroxybutyrate are not well understood. The main hypotheses relate to the lowering of gut pH resulting in the inhibition of the growth and virulence factor production of pathogenic bacteria^3^ and the direct delivery of energy to the PHB supplemented animals^21^. In addition to these, the possibility that the protective effect of PHB might be mediated by the induction of stress proteins, particularly Hsp70, has not been explored previously. In this *in vivo* study, our results provided conclusive evidence that PHB at a concentration of 100 mg/L induces the production of Hsp70 protein in *Vibrio*-challenged *Artemia* and interestingly, at this concentration, it also afforded complete protection to *Artemia* against *V. campbellii*. Previous studies using a rat model have demonstrated that butyrate selectively induced an important stress protein Hsp27 (but not Hsp70), both *in vitro* in cultured rat intestinal epithelial cells (IEC) and *in vivo* in the colon, and that this butyrate-induced expression of Hsp27 resulted in a greater cellular protection against oxidant injury^9^. In another *in vitro* study, Parhar et al. (2006) reported that butyrate not only induces Hsp25 but also regulates its phosphorylation in a rat IEC-18 crypt cell line and secondarily increased cellular resistance to apoptosis-inducing agents. From the results of the above-cited and present studies, it can be suggested that some of the protection offered by PHB in our model organism may be derived from the induction of Hsp70. It is worth mentioning that this induction was observed at the protein level but not at the mRNA level. The observed lack of correlation between h*sp70* mRNA and protein concentration can be explained by the different lifetimes of the molecules^24^: *hsp70* mRNA half-life is short (about 50 min) in cells after stress, even shorter in cells already containing Hsp70 protein^25^,^26^ and few copies per cell are produced, causing their concentrations in cells to fluctuate much more than those of the longer-lived (about 2 h) corresponding protein^27^. We must also acknowledge that the possible up-regulation of other stress proteins (like Hsp25, Hsp60, Hsp90) by PHB and their implication in conveying resistance to *Artemia* against *Vibrio* challenge likely exists as well^9^. Efforts are currently underway in our laboratory to unravel the effect of the Hsp inducer (PHB) on the expression of other various stress proteins in an attempt to address these possibilities.

Although the Hsp inducing effects of SCFAs are reported^9^,^23^,^28^, yet it is not clear how PHB or SCFAs induce stress proteins expression. At this point, we can argue that the PHB particles are (partially) degraded into different monomeric, dimeric and/or oligomeric forms in the *Artemia* gut and that this released fatty acid, through nonionic diffusion, might have caused cellular acidification. Lowering of cellular pH might have created a (mild) stress conditions that could have potentially driven the production of inducible Hsp70 in the intestinal epithelial cells^9^. However, this is pure speculation and needs further investigation.

As an invertebrate that lacks an acquired immune system, the brine shrimp *Artemia* depends on innate immune factors to build up resistance against pathogens. A growing body of evidence suggests that Hsp70 evokes protective responses in various animals (including in the aquaculture model organism *Artemia*) against bacterial diseases by inducing these defense factors^17^,^18^,^29^. Having demonstrated the effects of PHB on Hsp70 induction in *Artemia* host and in view of the association between Hsp70 and *Artemia* immune system, it is tempting to speculate whether PHB conveyed its protective effect through eliciting the *Artemia* defense system. To substantiate this hypothesis, we analyzed three important genes *proPO*, *ftn* and *tgase* encoding for immune effector proteins phenoloxidase, TGase and ferritin, respectively. The proPO system is composed, among other proteins, of the prophenoloxidase enzyme, which is the zymogen of phenoloxidase (PO)^30^,^31^. The PO induces its protective effect by its role in cuticular melanization, sclerotisation, wound healing, encapsulation and eventual killing of the pathogens^32^,^33^. Our results exhibited that the proPO system was markedly induced by PHB at a concentration of 100 mg/L. The induction effect was more prominent when *Artemia* were treated with PHB in conjunction with *Vibrio* compared to PHB alone, as indicated by elevation of the proPO mRNA transcript at 6 post challenge by 2.5-fold and PO activity at 6 and 12 h post challenge by 2-fold (see Figure 4A & 5). This result suggests an interaction effect of PHB and *Vibrio* on the induction of the proPO system in *Artemia*. Interestingly, this phenomenon correlates well with the Hsp70 protein induction by PHB at 100 mg/L concentration in the *Vibrio*-challenged group, suggesting that the induced Hsp70 might have accounted for eliciting the proPO immune system. In accordance with these findings, a previous study reported that induced Hsp70 coincides with increased expression of the *proPO* gene and its product PO enzyme in *Artemia* thereby promoting resistance to *V. campbellii* infection in the shrimp^17^.

In invertebrates with an open circulatory system, including *Artemia*, there is a need to quickly prevent the loss of blood or equivalent fluids through inflicted injuries (caused by pathogen) as these losses may have a marked impact on the survival of the invertebrate^34^. Also, there is a need to prevent microbes that have gained access to the body through the wound from disseminating throughout the open circulatory system. Therefore, many invertebrates possess a coagulation system to prevent such injuries from having too serious consequences. The defense molecule TGase is a major component of this system responsible for catalyzing the clotting reaction^35^,^36^. Our result revealed that PHB increased the expression of the *tgase* gene considerably shortly after challenge with *Vibrio* (markedly at 6 h and slightly at 12 h). This increase in the expression level did not appear to be statistically significant even though the increment was by about 2-fold (possibly due to high standard errors). In the *Artemia* that were subjected to only *Vibrio* challenge, no marked increase in the expression level of *tgase* was recorded at any of the tested time points. Interestingly, the results of *tgase* expression were supported by survival data especially in the group treated with 100 mg/L of PHB where more than 85% survival (compared to 11% in control) could be obtained. It is possible that the functional protein TGase encoded by the PHB-mediated *tgase* assists in host defense by preventing tissue damage and simultaneously by blocking (further) progression of *Vibrio* infection in *Artemia*. Similar observations have been reported by Babu et al. (2013)^37^, where shrimp fed a diet containing immunostimulants showed significantly higher expression of *tgase* and had higher protection against the shrimp pathogen white spot syndrome virus.

Iron has been considered as an essential element required for the survival and growth of most organisms, both hosts and pathogens^38^. It has almost been certain that tight regulation of iron (regarded as double-edged sword) is a paramount defense mechanism of the host^39^. For multiplying and inducing pathogenic effects within the host, the pathogen competes for the host's iron^40^. A variety of iron-withholding defense mechanisms are being used by the host to limit the availability of essential iron from the bacteria without causing deficiency for itself. In this study, we focused on the *ferritin* (*ftn*) immune gene encoding for protein ferritin, known to play crucial role as buffer against iron deficiency and iron overload^39^. Our results revealed that PHB did not up-regulate *ftn* transcription during *Vibrio* infection as the expression level was similar to that in the infected/uninfected *Artemia*. In contrast, in the later period of *Vibrio* infection (24 h), the *ftn* expression level was relatively low in the PHB-treated groups. As a major iron-binding protein, it is conceivable that ferritin is a rich nutrient resource and it would be an obvious target for bacteria to pirate iron from^39^. Unaltered or reduced expression of *ftn* gene (and presumably in the ferritin protein level) in the PHB-treated group challenged with *Vibrio* could be a defensive strategy of the host to deprive iron from the bacteria^39^.

Another interesting observation that was noted in this study was that PHB above a threshold concentration (> 100 mg/L) had an adverse effect upon *Vibrio*-challenged *Artemia* as survival of *Vibrio*-challenged *Artemia* decreased with PHB concentrations increasing above 100 mg/L. This effect was also associated with a significant decrease in the Hsp70 level in *Vibrio*-challenged *Artemia* treated with high PHB dose (1000 mg/L, data not shown). As such toxicity is to be expected, even a benign compound would become toxic after a certain threshold concentration^37^. However, in this study, it is clear that the effective dose of this compound was 10 times lower than the toxic dose (and through further compound optimization i.e., by the addition of PHB degrading bacteria, this effective dose could possibly be further lowered^7^). This indicates that PHB has a good safety margin and therefore appeared to be safe for therapeutic use in (aquaculture) animal production sector. A possible explanation for the adverse effects of PHB at high dose could be due to the fact that at higher PHB concentrations, the pH in the *Artemia* gut might have dropped down remarkably, which could likely contribute to PHB toxicity and thus, more Hsp70 may be utilized to reverse the (deleterious) effect induced by a combination of PHB toxicity and *V. campbellii* insults than by the latter alone^23^,^41^.

In essence, the results presented here provide new insights on the polymer of ß-hydroxybutyrate, PHB, as a novel inducer of Hsp70 biosynthesis under the described experimental conditions. Our findings also imply that this compound-mediated Hsp70 seems to be responsible for generating protective innate immunity through regulating the expression of *proPO*, *tgase* and *ftn* immune genes in the *Artemia* larvae, offering new clues for the mechanism of the protective effect of PHB against pathogenic *V. campbellii*. Combining the present study with its outstanding protective effect reported previously, PHB therefore can be a potent bio-control agent in different host–microbe systems, such as for instance *Salmonella* infections in poultry and swine or bacterial infections in other crustaceans and fishes. To gain more insights into the functional properties of PHB, more detailed studies should be carried out in future by employing RNAi techniques to knock-out Hsp70 gene and determining the effect of PHB treatment on the host innate immune response by performing time-dependent micro-array analysis.

## Methods

### Axenic hatching of *Artemia*

All challenge tests were performed with high quality hatching cysts of *Artemia fransciscana* originating from the Great Salt Lake, Utah, USA (INVE Aquaculture, Dendormonde, Belgium) as described previously^42^. Briefly, *Artemia* (2.5 g) cysts were hydrated in 89 mL of distilled water for 1 h. Sterile cysts and larvae were obtained via decapsulation using 3.3 mL NaOH (32%) and 50 mL NaOCl (50%). All manipulations were carried out under a laminar flow hood and all tools were sterilized. The decapsulation was stopped after about 2 min by adding 50 mL Na_2_S_2_O_3_ at 10 g/L. The decapsulated cysts were washed with sterile seawater containing 35 g/L of instant ocean synthetic sea salt (Aquarium Systems, Sarrebourg, France). The cysts were suspended in 1-L glass bottles containing sterile seawater and placed in rectangular tank containing water maintained at 28°C using a thermostatic heater for incubation for 18–20 h with constant illumination of approximately 2000 lux. After 20 h of incubation, the axenicity of the *Artemia* larvae was verified by spread plating 100 mL of the hatching water on Marine Agar (Difco, Detroit, USA) followed by incubating at 28°C for 5 days^43^. Experiments started with non-sterile larvae were discarded.

### Bacterial strains for *in vivo* challenge tests

Two bacterial strains were in this study: LVS3 (*Aeromonas hydrophila*) and *Vibrio campbellii* LMG21363. LVS3 (autoclaved) were used as feed for *Artemia* and *V. campbellii* as a pathogen for the challenge assay*.* Both the strains were stored in 40% glycerol at −80°C. 10 µL of these stored cultures were inoculated into fresh Marine Broth (Difco Laboratories, Detroit, USA) and incubated overnight at 28°C under constant agitation. The grown LVS3 culture was autoclaved, washed in autoclaved artificial seawater and added to the *Artemia* culture water at approximately 10^7^ cells/mL. Live *V. campbellii* were also added to the *Artemia* culture water in a similar fashion at 10^7^ cells/mL concentrations.

### *Artemia* challenge assay

Challenge tests were performed as described previously^16^, with slight modifications. Briefly, after hatching, groups of 30 larvae were transferred to sterile 40 mL glass tubes that contained 30 mL of sterile artificial seawater. The tubes were inoculated with *V. campbellii* and the *Artemia* therein were fed with autoclaved LVS3. PHB particles of 25–30 µm size were added to the *Artemia* culture water at different concentrations (10, 100, 250, 500 and 1000 mg/L). After feeding and the addition of the appropriate compound and/or bacteria, the glass tubes were put back on the rotor and kept at 28°C. The survival of *Artemia* was scored 2 days after the addition of the pathogen. All manipulations were done under a laminar flow hood in order to maintain gnotobiotic conditions of the cysts and nauplii. Each treatment was done in quintuplicate.

### Sampling and analysis

After 20 h of incubation at 28°C, swimming *Artemia* larvae were collected, counted volumetrically and transferred to 500 mL sterile glass bottles. The larvae were treated with the dose of PHB that gave the best protection in the *Artemia* challenge assay and were simultaneously fed and challenged with autoclaved LVS3 and *V. campbellii*, respectively, as described above. Each treatment was carried out in triplicate. *Artemia* samples containing live larvae (0.1 g and 60 mg for analysis of protein and genes, respectively) were sampled from all treatments at 6, 12 and 24 h of *Vibrio* exposure, rinsed in cold distilled water, immediately frozen in liquid nitrogen and then stored at −80°C until further analysis (see the following headings).

### Protein extraction and Hsp70 analysis

The *Artemia* samples were homogenized in cold buffer K (150 mM sorbitol, 70 mM potassium gluconate, 5 mM MgCl_2_, 5 mM NaH_2_PO4, 40 mM HEPES, pH 7.4)^44^, and supplemented with protease inhibitor cocktail (Sigma-Aldrich, USA) as recommended by the manufacturer. Subsequent to centrifugation at 2200 × *g* for 1 min at 4°C, supernatant protein concentrations were determined by the Bradford method using bovine serum albumin as standard^45^. Supernatant samples were then combined with loading buffer, vortexed, heated at 95°C for 5 min and electrophoresed in 10% SDS-PAGE gels, with each lane receiving equivalent amounts of protein. HeLa (heat shocked) cells (Enzo Life Sciences, USA) (6 µg) were loaded on to one well to serve as a positive control and for calculating the amount of Hsp70 in the sample. Gels were then transferred to polyvinylidene fluoride membranes (BioRad Immun-BlotTM PVDF) for antibody probing. Membranes were incubated with blocking buffer [50 mL of 1x phosphate buffered saline containing 0.2% (v/v) Tween-20 and 5% (w/v) bovine serum albumin] for 60 min at room temperature and then with mouse monoclonal anti-Hsp70 antibody, clone 3A3 (Affinity BioReagents Inc., Golden, CO), which recognizes both constitutive (heat shock cognate, Hsc70) and inducible Hsp70^43^, at the recommended dilution of 1:5000. Horseradish peroxidase conjugated donkey anti-mouse IgG was used as secondary antibody at the recommended dilution of 1:2500 (Affinity BioReagents Inc., Golden, CO). The membranes were then treated with enhanced chemiluminescence reagent (GE healthcare, UK) and the signals were detected by a ChemiDoc MP Imaging System (Biorad, Belgium). The relative signal intensity was quantified by densitometry with Biorad Image Lab™ Software version 4.1.

### Assay of heat shock protein 70 (*hsp70*), prophenoloxidase (*proPO*), transglutaminase (*tgase*) and ferritin (*ftn*) gene expression by quantitative real-time PCR (qPCR) analysis

Total RNA was extracted from the *Artemia* samples using the SV total RNA isolation kit (Promega, Belgium) according to the manufacturer's instructions, after which the RNA was quantified spectrophotometrically (NanoDrop Technologies, Wilmington, DE, USA). First strand cDNA was synthesized from 2 µg total RNA using the RevertAid™ H minus First strand cDNA synthesis kit (Fermentas Gmbh, Germany) according to the manufacturer's instructions. The expression of *hsp70*, *proPO, tgase* and *ftn* genes in *Artemia* was analyzed by qPCR using a pair of specific primers^15^. The qPCR amplifications were carried out in a total volume of 25 µL, containing 5.5 µL of nuclease free water, 1 µL of each primer, 12.5 µL of Maxima SYBR Green qPCR Master mix (Fermentas, Cambridgeshire) and 5 µL of cDNA template. The qPCR was performed in a One Step qPCR instrument (Applied Biosystems) using a four-step amplification protocol: initial denaturation (10 min at 95°C); 40 cycles of amplification and quantification (15 s at 95°C, 30 s at 60°C, and 30 s at 72°C); melting curve (55–95°C with a heating rate of 0.10°C s^−1^ and a continuous fluorescence measurement) and cooling (4°C). The β-actin gene was used as a reference gene. Master mixes were prepared in duplicate for each sample and qPCR for target and reference genes was performed. Relative quantification of target gene transcripts with a chosen reference gene transcript was done following the Pfaffl method with the Relative Expression Software tool (REST^©^) as described previously^46^.

### Assay of phenoloxidase (PO) activity

For PO assay, *Artemia* samples (0.1 g) were collected after 6, 12 and 24 h of *Vibrio* challenge from all the treatments. Protein extracts were prepared from sampled larvae^16^ and their PO activity was determined according to Ashida et al. (1983)^47^ with some modification. Equal volumes and protein amounts of each extract were added to the wells of 24-well microtitre plates to which 1 mL of the substrate L-DOPA (0.5 mM) dissolved in 100 mM sodium acetate-citric acid buffer (pH 7.1) containing 10 mM CaCl_2_, was added. The reaction mixture was incubated in the dark at 30°C for 48 h and OD was measured at 490 nm using an ELISA reader (Tecan, Männedorf, Switzerland). Increases in OD_490_ due to spontaneous non-enzymatic dopachrome production were determined in wells without protein extract. Apparent PO activity was recorded as the change in absorbance over 48 h and it was expressed in units as defined previously^16^. The protein concentration of the extracts was measured by the Bradford method.

### Statistical analysis

Survival data were arcsin transformed to satisfy normality and homocedasticity requirements as necessary. All the data were then subjected to one-way analysis of variances followed by Duncan's multiple range tests using the statistical software Statistical Package for the Social Sciences version 14.0 to determine significant differences among treatments. Results for target gene mRNA quantification are presented as fold expression relative to *Artemia* actin. The expression level in control was regarded as 1.000 and thereby the expression ratio of the treatments was expressed in relation to the control. Significant differences in expression between control and treatments were analyzed by Relative Expression Software tool–Multiple condition solver (REST–MCS) Version 2 using Pair Wise Fixed Reallocation Randomization Test^©^^43^. Significance level was set at P < 0.05.

## Author Contributions

K.B. conceived, designed and wrote the manuscript. T.T.H., P.N., Y.N. and S.K.G. carried out the experiments and performed the analyses. P.D.S. and P.B. assisted in the experimental design, and preparation of the manuscript.

## Supplementary Material

Supplementary Information Supplementary Figure S1
