# Supplementary material for: Probing the protective mechanism of poly-ß-hydroxybutyrate against vibriosis by using gnotobiotic Artemia franciscana and Vibrio campbellii as host-pathogen model
Source: Sci Rep. 2015 Mar 30;5:9427. doi: 10.1038/srep09427 (PMC4378509; doi:10.1038/srep09427)
Supplement: Supplementary Information — Supplementary Figure S1 [file srep09427-s1.pdf]

Supplementary information:

Probing the protective mechanism of poly-ß-hydroxybutyrate against vibriosis by using gnotobiotic *Artemia franciscana* and *Vibrio campbellii* as host-pathogen model

Kartik Baruah[1], Tran T. Huy[1], Parisa Norouzitallab[1], Yufeng Niu[1], Sanjay K. Gupta[2], Peter De Schryver[1] & Peter Bossier[1]

[1]Lab of Aquaculture & *Artemia* Reference Center, Department of Animal Production, Faculty of Bioscience Engineering, Ghent University, Rozier 44, Gent 9000, Belgium, [2]Directorate of Cold Water Fisheries Research, Chhirapani Field Centre, Champawat, Uttarakhand 262523, India.

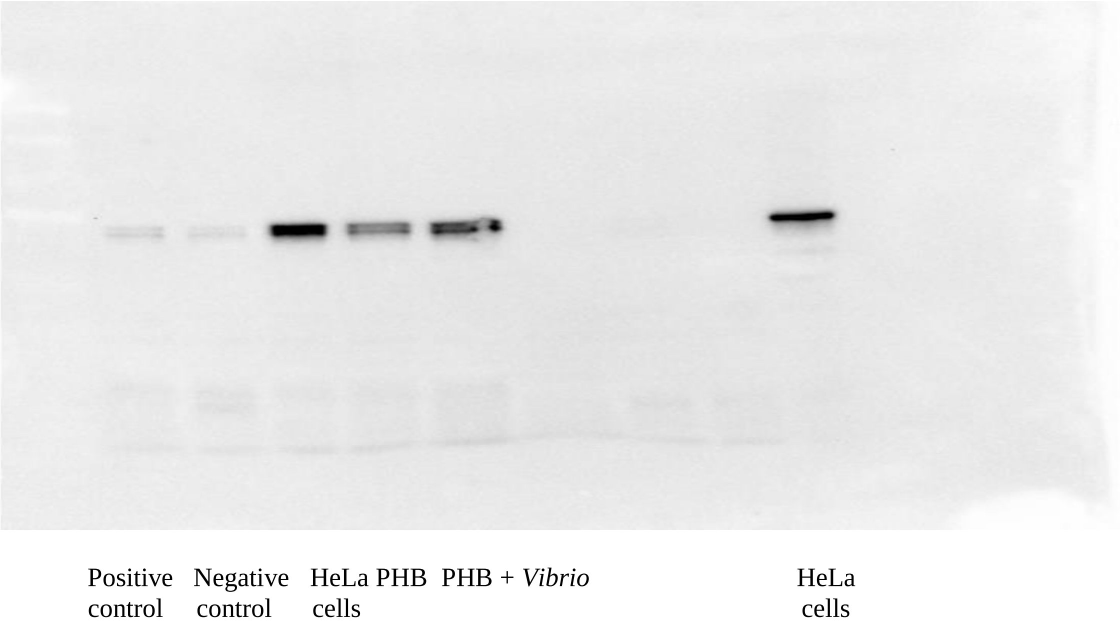

Figure S1 | Uncropped immunoblot image showing that PHB at an optimum concentration induces Hsp70 production in *Artemia* larvae challenged with *V. campbellii*. For the treatment groups, refer to Fig. 2. for explanation. *Artemia* samples were collected for analysis of Hsp70 induction at 6 h of *Vibrio* challenge. Protein extracted from different groups was resolved in SDS-PAGE gel and then transferred to polyvinylidene fluoride membranes and probed with antibody to *Artemia* Hsp70. Seven microgram of *Artemia* protein was loaded

in each lane. HeLa (heat shocked) cells (6 µg) were loaded on to one well to serve as a positive technical control and for calculating the relative amount of Hsp70 in the sample. (B) Quantitative analysis of Hsp70 in the *Artemia* larvae (expressed relative to the amount in HeLa cells).
